# Highly thermal stable RNase A@PbS/ZnS quantum dots as NIR-IIb image contrast for visualizing temporal changes of microvasculature remodeling in flap

**DOI:** 10.1186/s12951-022-01312-0

**Published:** 2022-03-12

**Authors:** Yimeng Yang, Mo Chen, Peng Wang, Liman Sai, Chen Chen, Pingkang Qian, Shixian Dong, Sijia Feng, Xing Yang, Hao Wang, Amr M. Abdou, Yunxia Li, Shiyi Chen, Yuefeng Hao, Dongling Ma, Shaoqing Feng, Jun Chen

**Affiliations:** 1 Sports Medicine Institute of Fudan Univerisity, Department of Sports Medicine and Arthroscopy, Huashan Hospital, Fudan University, Shanghai, 200040 China; 2 Department of Physics, Shanghai Normal University, Guilin Road 100, Shanghai, 200234 China; 3 Department of Sports Medicine, Shanghai Sixth People’s Hospital Affiliated to Shanghai Jiao Tong University, Shanghai, 200233 China; 4 Department of Orthopedics, Kunshan Hospital of Traditional Chinese Medicine, West Chaoyang Road, Kunshan, 215300 Jiangsu China; 5 Department of Anatomy and Physiology, School of Medicine, Shanghai Jiao Tong University, Shanghai, 200025 China; 6 Department of Orthopedics, Affiliated Suzhou Hospital of Nanjing Medical University, Suzhou, 215500 Jiangsu China; 7 Asia Cellular Therapeutics (Shanghai) Co., Ltd, Shanghai, 201499 China; 8 Department of Microbiology and Immunology, National Research Center, Giza, Egypt; 9 Institut National de la Recherche Scientifique Centre Énergie Matériaux et Télécommunications, Université du Québec, 1650 Boulevard Lionel-Boulet, Varennes, QC J3X 1S2 Canada; 10 Department of Plastic and Reconstructive Surgery, Shanghai Ninth People’s Hospital, School of Medicine, Shanghai Jiao Tong University, Shanghai, 200011 China

**Keywords:** Thermal stability, Vascular imaging, Quantum dots, NIR-IIb window, Intraoperative imaging navigation

## Abstract

Surgeons face great challenges in acquiring high-performance imaging because fluorescence probes with desired thermal stability remains rare. Here, hybrid lead sulfide/zinc sulfide quantum dots (PbS/ZnS QDs) nanostructures emitting in the long-wavelength end of the second near-infrared (NIR-IIb) window were synthesized and conjugated with Ribonuclease-A (RNase A). Such formed RNase A@PbS/ZnS QDs exhibited strong NIR IIb fluorescence and thermal stability, as supported by the photoluminescent emission assessment at different temperatures. This will allow the RNase A@PbS/ZnS QDs to provide stable fluorescence signals for long-time intraoperative imaging navigation, despite often happened, undesirable thermal accumulation in vivo. Compared to NIR-IIa fluorescence imaging, NIR-IIb vascular fluorescence imaging achieved larger penetration depth, higher signal/background ratios and nearly zero endogenous tissue autofluorescence. Moreover, these QDs illustrate the reliability during the real-time and long-time precise assessment of flap perfusion by clearly visualizing microvasculature map. These findings contribute to intraoperative imaging navigation with higher precision and lower risk.

**Graphical Abstract:**

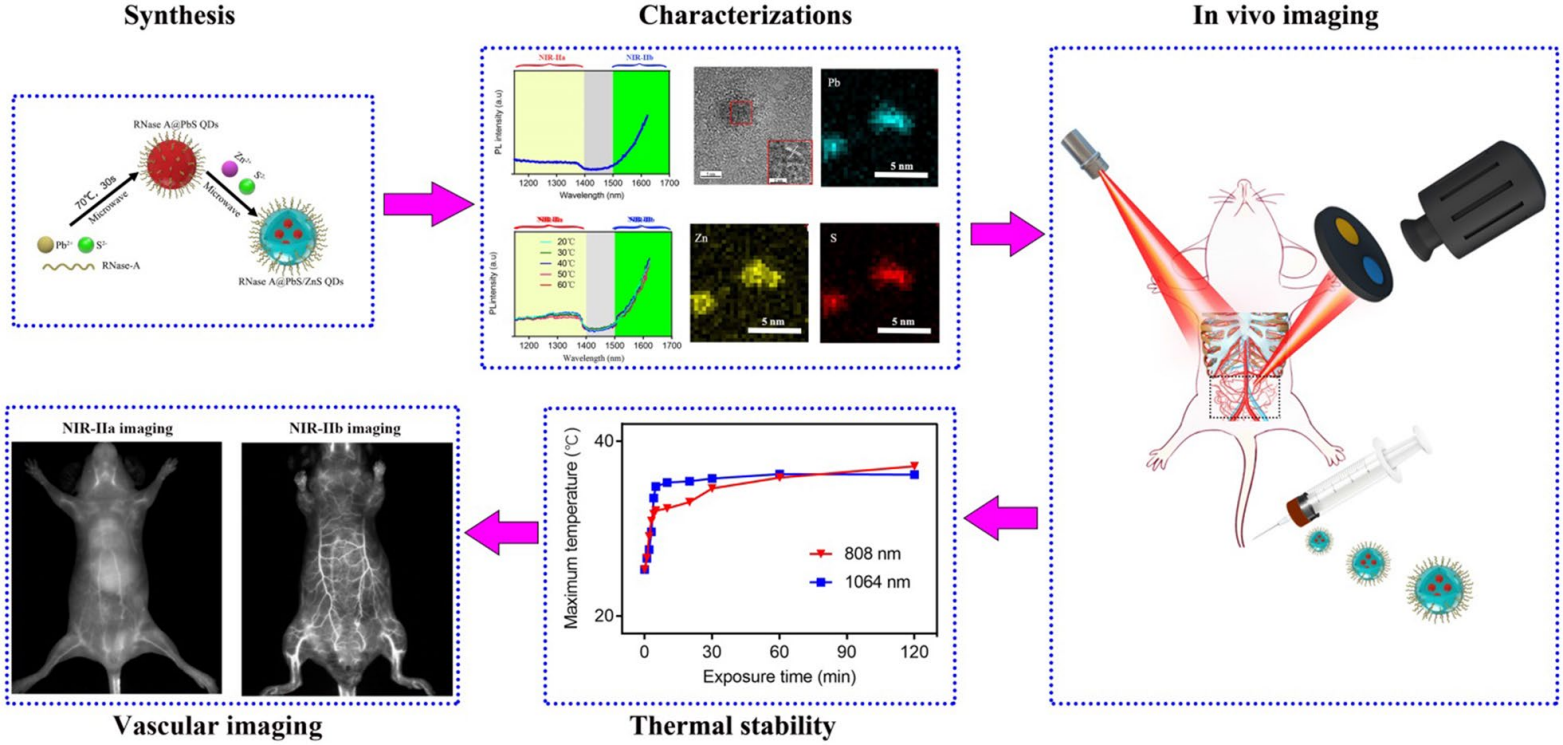

**Supplementary Information:**

The online version contains supplementary material available at 10.1186/s12951-022-01312-0.

## Introduction

Fluorescence imaging-guided surgery has gained recognition as a powerful technique and is expected to provide surgeons with real-time visual guidance of pathological tissues that are otherwise invisible to the naked eye [1–3]. However, conventional intraoperative fluorescent imaging have been hampered by low spatial resolution, limited penetration depth and low signal/background ratios (SBR) due to the strong photon scattering and autofluorescence background arising from biological tissues in the visible range (400–700 nm) and the first near-infrared (NIR-I, 700–900 nm) window [4]. In this context, fluorescent imaging in the state-of-the-art second near-infrared region (NIR-II, 1000–1700 nm; NIR-IIa, 1000–1400 nm; NIR-IIb, 1500–1700 nm), has attracted great interest as it affords significantly higher spatial resolution and deeper tissue penetration owing to suppressed tissue scattering and negligible autofluorescence background [1, 5–11]. In particular, NIR-IIb emitters not only avoid water absorption overtone peaks (1450 nm) to minimize the attenuation of the fluorescence signal, but also provide the lowest photon scattering in the entire NIR-II window to achieve the highest spatial resolution (approximately micrometers) and nearly zero endogenous biological imaging [12, 13]. Although the light scattering is further reduced when imaging above 1700 nm, the water absorption rate increases and the sensitivity of the commonly used InGaAs detector decreases, which lead to unsatisfactory imaging performance [12]. Thus, NIR-IIb fluorescence imaging holds the greatest promise for biological imaging by achieving both improved penetration depth and imaging resolution.

So far, several probes have been developed as candidates for in vivo NIR-IIb fluorescence imaging, including single-walled carbon nanotubes [13], rare earth nanoparticles [9, 14–17], organic dyes [18, 19], and inorganic quantum dots (QDs) [12, 20, 21]. However, the necessity of external excitation light during intraoperative fluorescent image-guided surgery inevitably leads to light-induced heating effect [22]. In particular, surgical operation requires from scores of minutes to hours, and laser irradiation during this time period could heat up the fluorescence probes and surrounding tissues [23]. The thermal accumulation in biological tissue could be a critical issue as the optical property of fluorescence probes and imaging performance can be largely degenerated by undesirable local heating effect [24, 25]. Clearly, thermally stable probes emitting in the NIR-IIb window are in urgent demand, driving the development of alternative probes for intraoperative NIR-IIb fluorescence imaging and rendering the undesirable local heating effect less of a concern.

Previously, we demonstrated that Ribonuclease-A (RNase A) can template the synthesis of highly fluorescent QDs and effectively reduced their cytotoxicity [26, 27]. Moreover, RNase A allows for significantly reduced thermal quenching to stabilize QDs due to its outstanding thermal stability, which can sustain 100 °C without aggregation [26]. Therefore, the RNase A assisted synthesis approach was extended to assist the formation of hybrid lead sulfide/zinc sulfide quantum dots (PbS/ZnS QDs) in the current work. Here, we successfully developed a novel NIR-IIb nanoprobe (RNase A@PbS/ZnS QDs, emitting beyond 1500 nm), which was proved to exhibit excellent photostability and salient thermal stability. Local heating does not weaken the fluorescence signal of RNase A@PbS/ZnS QDs, displaying their impressive potential in intraoperative fluorescence image-guided surgery that requires continuous laser irradiation. Compared to NIR-IIa fluorescence imaging, NIR-IIb vascular fluorescence imaging achieved larger penetration depth and higher SBR with nearly zero endogenous tissue autofluorescence. The pharmacokinetics and histomorphometric analysis further indicates the low cytotoxicity of these QDs. Furthermore, the NIR-IIb fluorescence imaging offers a favorable approach for intraoperatively dynamic assessment of the area of flap perfusion, suggesting the possibility of providing a more accurate prediction in intraoperative fluorescence image-guided surgery for flap transplantation.

## Results and discussion

**Fig. 1:**
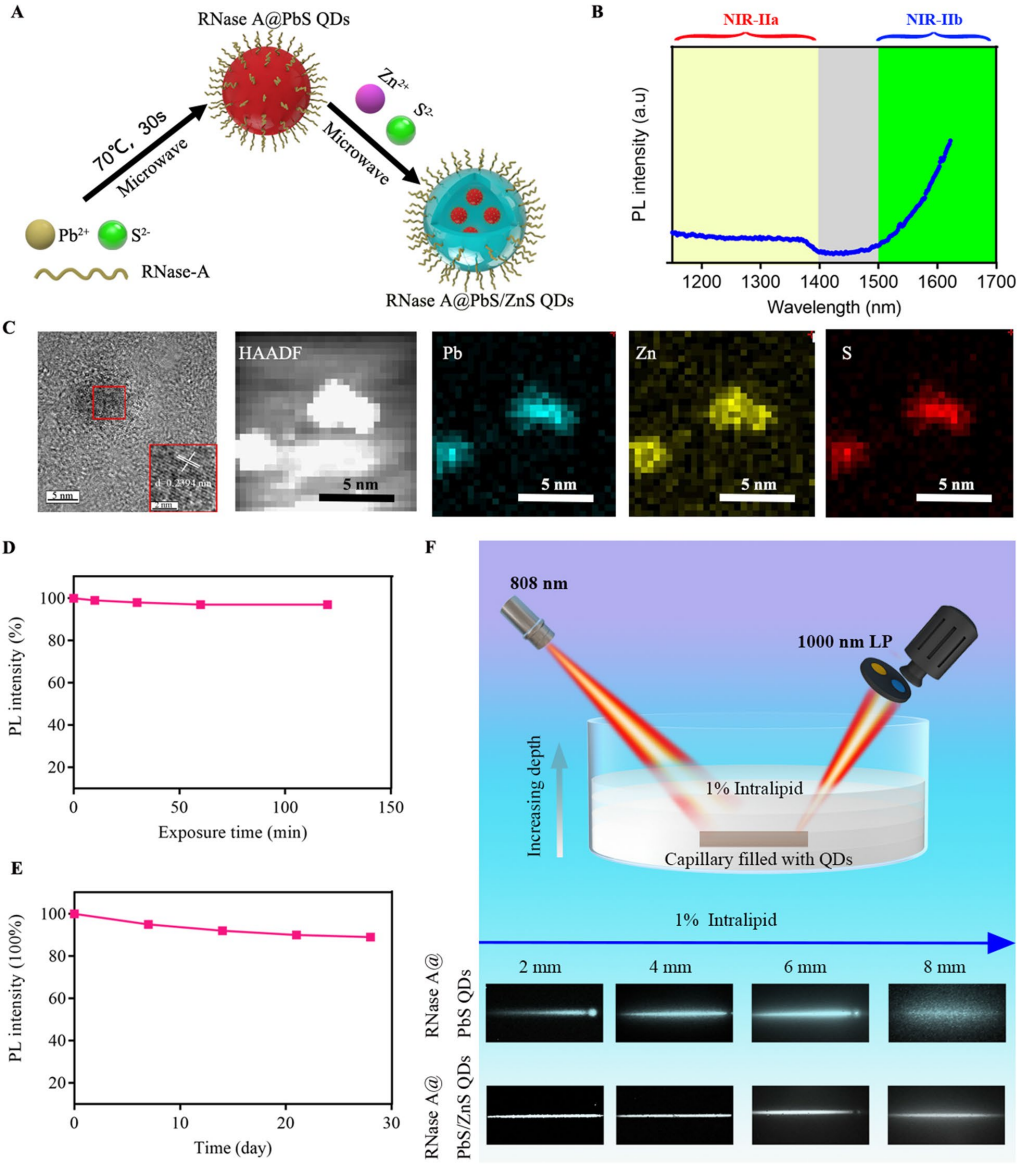
Characterization and optical properties of NIR-IIb emitting RNase A@PbS/ZnS QDs. **A** Schematic design of RNase A@PbS/ZnS QDs. **B** Fluorescence emission spectrum of RNase A@PbS/ZnS QDs. **C** High-resolution TEM image and HAADF-STEM images of as-prepared RNase A@PbS/ZnS QDs. The energy-dispersive X-ray spectroscopy elemental maps showed the distribution of Pb (blue), Zn (yellow), and S (red). Scale bar: 5 nm. **D** Photostability of RNase A@PbS/ZnS QDs under continuous 808-nm laser exposure for 2 h. **E** The long-time stability of RNase A@PbS/ZnS QDs stored at room temperature (20–25 °C) over the course of 4 weeks. **F** Fluorescence images of capillary tubes filled with PbS QDs or RNase A@PbS/ZnS QDs immersed at varied depths in 1% Intralipid (LP, long-pass). These results confirmed the excellent fluorescence properties and stability of RNase A@PbS/ZnS QDs, which suggested the superior fluorescence imaging potential in the NIR-IIb region

The synthesis of RNase A@PbS/ZnS QDs involved the formation of a PbS QD (λ_em_ = 1300 nm) under microwave (70 °C, 30 s) in aqueous solution at first [27], and then the formation with a ZnS structure under microwave (70 °C, 60 s) (Fig. 1A). After forming the ZnS structure, the photoluminescent (PL) emission of RNase A@PbS/ZnS QDs was measured and the result showed that the PL band of as-prepared RNase A@PbS/ZnS QDs have red-shifted to the NIR-IIb region with a maximum PL beyond 1600 nm (Fig. 1B). By using IR-26 as a reference [27], the quantum yield of the RNase A@PbS/ZnS QDs was determined to be ~ 10.1%. The UV–vis spectrum of the QDs showed a characteristic peak of RNase-A at 280 nm (Additional file 1: Fig. S1A). The as-prepared RNase A@PbS/ZnS QDs were further characterized by transmission electron microscopy (TEM). The successful formation of ZnS structure was evidenced by elemental mapping using high-angle annular dark-field scanning TEM (HAADF-STEM) (Fig. 1C) and energy-dispersive X-ray (Additional file 1: Fig. S1B). Additional file 1: Fig. S1C showed the corresponding selected area electron diffraction. The as-prepared RNase A@PbS/ZnS QDs were monodisperse with a diameter of 4.9 ± 0.3 nm (Additional file 1: Fig. S1D, E).

Besides, we characterized the optical stability of RNase A@PbS/ZnS QDs under continuous illumination. The photobleaching experiment showed that even after 120 min of irradiation with an 808 nm diode laser, the RNase A@PbS/ZnS QDs retained about 96% of their initial photoluminescence without significant further decay (Fig. 1D). As current image-guided diagnosis and surgeries require continuous monitoring of dynamic physical processes, the superior anti-photobleaching of RNase A@PbS/ZnS QDs renders them convenient and appealing for clinical application. Besides, after being stored at room temperature (20–25 °C) for 4 weeks, the RNase A@PbS/ZnS QDs still kept their strong brightness without obvious decay in terms of PL intensity (Fig. 1E). Moreover, the effect of an absorbing and scattering medium on fluorescence properties has been of great significance in biomedical imaging. Therefore, a tissue phantom imaging study in the NIR-IIa and NIR-IIb regions using 1% Intralipid as a mimic of biological tissue was performed to compare the clarity and penetration depth of PbS QDs and RNase A@PbS/ZnS QDs. As shown in Fig. 1F, a capillary tube filled with PbS QDs or RNase A@PbS/ZnS QDs was immersed in the 1% Intralipid at various depths. With increasing depth, there was a loss of image clarity and broadening of imaging feature width due to the enhanced scattering. When the capillary tubes were close to the surface (depth = 2 mm), sharp images were obtained for both cases. However, the image of the capillary tube filled with PbS QDs was more significantly broadened with the increase of depth due to more serious scattering. By contrast, a sharp edge of the capillary tube filled with RNase A@PbS/ZnS QDs could be maintained at a penetration depth of 8 mm, which was attributed to minimized scattering. The phantom study indicated that NIR-IIb imaging could afford better spatial resolution at deeper tissue penetration owing to minimized scattering [6, 7]. These results suggest that RNase A@PbS/ZnS QDs with stable NIR-IIb fluorescence signals and reduced tissue scattering were suitable for in vivo deep tissue biomedical applications. To characterize the cytotoxicity of RNase A@PbS/ZnS QDs on mesenchymal stem cells (MSCs), the live/dead staining assay was performed. The results demonstrated that most of the cells incubated with different concentrations of RNase A@PbS/ZnS QDs were alive (Additional file 1: Fig. S2a). Furthermore, MSCs treated with different concentrations, even with a high concentration of 30 µg/mL, showed no significant differences in MSC morphology or cell viabilities (P > 0.05, Additional file 1: Fig. S2b).

**Fig. 2:**
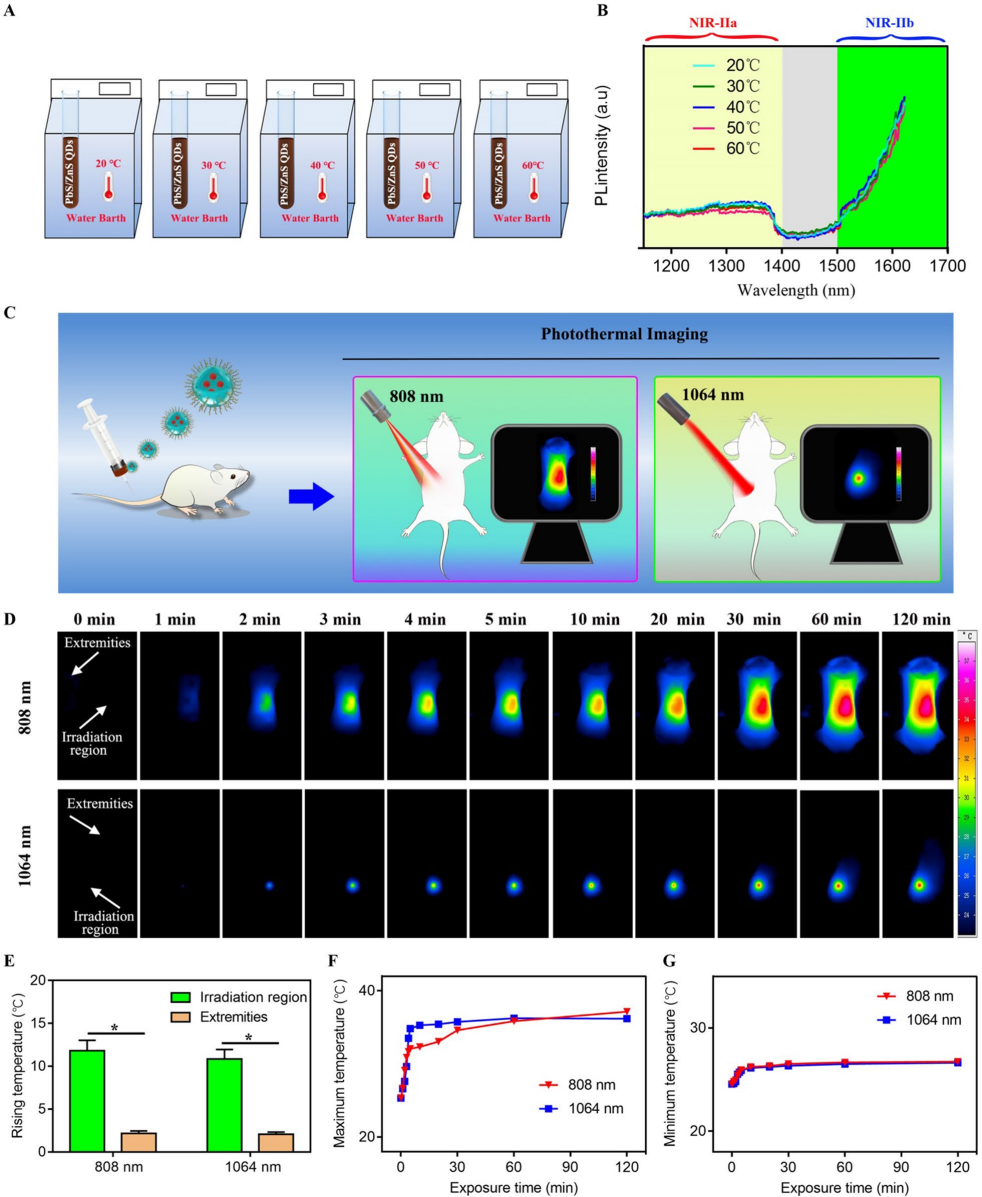
Assessment of the thermal stability of RNase A@PbS/ZnS QDs. **A** Schematic illustration of the in vitro study protocol. **B** The PL spectra of RNase A@PbS/ZnS QDs solution at different temperatures. **C** Schematic illustration of the in vivo study protocol on living mice. **D** Thermal images of mice under irradiation of 808 nm or 1064 nm after systemic administration of RNase A@PbS/ZnS QDs. **E** Rising temperature at the irradiation region or the extremities under irradiation of 808 nm or 1064 nm. **F** Maximum temperature as a function of laser irradiation time after systemic administration of RNase A@PbS/ZnS QDs. **G** Minimum temperature as a function of laser irradiation time after systemic administration of RNase A@PbS/ZnS QDs. * indicated P < 0.05

Biological tissue is a remarkable collection of endogenous biological molecules, many of which, such as haemoglobins, melanins, aromatic amino acid residues in proteins, reduced nicotinamide adenine dinucleotide and heterocyclic flavins, are chromophores that turn excitation and/or fluorescence photons into heat dissipation [1]. It should be noted that laser-induced tissue heating is not the only undesirable thermal effect upon irradiation (named tissue-heating); fluorescence probes can also be an efficient light absorber that can convert absorbed photon energy into heat (named probe-heating). The undesirable thermal accumulation might cause fluorescence quenching in the NIR-II biological imaging [22]. Thus it is essential to consider and assess the thermal stability of fluorescence probes in order to control local heating in a proper range. Then the PL emission of RNase A@PbS/ZnS QDs was examined under different temperature conditions (Fig. 2A). As shown in Fig. 2B, neither fluorescence intensity nor peak wavelength shift was observed for the RNase A@PbS/ZnS QDs kept at different temperatures from 20℃ to 60℃. Encouraged by the thermal stability of the RNase A@PbS/ZnS QDs measured in vitro, the thermal property of RNase A@PbS/ZnS QDs under 808 nm irradiation and 1064 nm irradiation was further investigated on the living body (Fig. 2C). As shown in Fig. 2D, the temperature variation of the mouse under 808 nm irradiation was similar to that under 1064 nm irradiation. The temperature distribution within the abdominal region gradually decreased from irradiation region to periphery. It could be seen that the temperature decreased significantly as it moved away from the irradiation region and the temperature at the far end of the extremities was only ≈ 71% of that at the irradiation region.

Additionally, the rising temperature at the irradiation region was nearly five times more than that at the extremities after 120 min of irradiation (Fig. 2E). It is proposed that the rising temperature at the irradiation region might be a result of the tissue-heating and probe-heating, while the rising temperature at the extremities was mainly due to the probe-heating. The temperature at the irradiation region under 1064 nm irradiation increased more rapidly than that under 808 nm irradiation. One reason may be that the diameter of the 808 nm laser outlet was 5–8 mm, which was larger than that of 1064 nm laser (1.5 mm) while the power of 808 nm irradiation (0.2 W/cm^2^) was lower than that of 1064 nm irradiation (0.25 W/cm^2^). Furthermore, the temperature of the mice during continuous laser irradiation for 2 h, regardless of 808 nm or 1064 nm illumination, is in the 20–60 °C temperature range (Fig. 2G), suggesting that the laser-induced thermal accumulation will not change the fluorescence property of RNase A@PbS/ZnS QDs based on the in vitro results (Fig. 2B). Taken all together, the results validated the thermal stability of the RNase A@PbS/ZnS QDs for in vivo long time biological imaging.

**Fig. 3:**
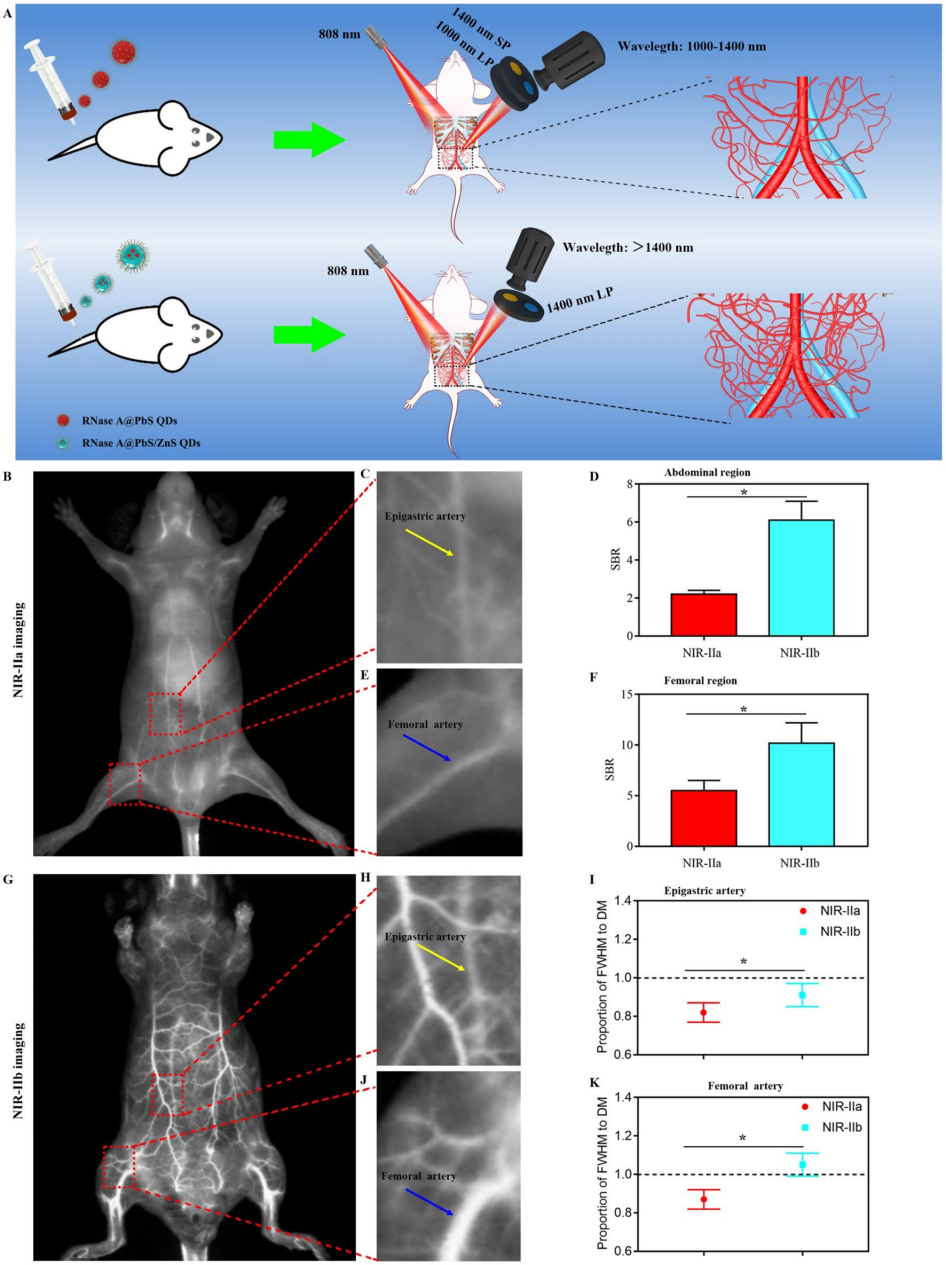
Comparison of whole-body vascular imaging between NIR-IIa window and NIR-IIb window. **A** Schematic illustration of the NIR-IIa imaging and NIR-IIb imaging strategy. **B** NIR-IIa vascular imaging for whole-body imaging of living mice. **C** Region of interest (abdominal region) from the red-dashed box in **B**; the yellow arrows indicated the epigastric artery. **D** The quantified SBR of abdominal region in NIR-IIa imaging and NIR-IIb imaging. * indicated P < 0.05. **E** Region of interest (femoral region) from the red-dashed box in **B**; the blue arrows indicated the femoral artery. **F** The quantified SBR of femoral region in NIR-IIa imaging and NIR-IIb imaging. * indicated P < 0.05. **G** NIR-IIb vascular imaging for whole-body imaging of living mice. **H** Region of interest (abdominal region) from the red-dashed box in **G**; the yellow arrows indicated the epigastric artery. **I** The epigastric artery diameter proportion of FWHM to DM. * indicated P < 0.05. **J** Region of interest (femoral region) from the red-dashed box in **G**; the blue arrows indicated the femoral artery. **K** The femoral artery diameter proportion of FWHM to DM. * indicated P < 0.05

Having demonstrated the excellent thermal stability of RNase A@PbS/ZnS QDs, we next compared the imaging performance of NIR-IIa imaging and NIR-IIb imaging in vivo. High-resolution and non-invasive vascular imaging enables direct visualization and real-time feedback of dynamic changes of pathological process and monitoring hemodynamic information. Thus, the RNase A@PbS/ZnS QDs were intravenously injected into a mouse’s tail vein and its angiography was recorded by an InGaAs camera with two long-pass (LP) filters (1000 nm and 1500 nm) and one short-pass (SP) filter (1400 nm) (Fig. 3A). After intravenous injection, the whole vessel network of the mouse was clearly visualized (Fig. 3B, G). As compared with the NIR-IIa imaging, the NIR-IIb imaging exhibited superior resolution with an approximately transparent background. In detail, the major blood vessels (epigastric artery, Fig. 3C; femoral artery, Fig. 3E) and its branch vessels could be unambiguously distinguished from the brighter surrounding background tissue in the NIR-IIa imaging, but more than that, the NIR-IIb imaging offered a clear vascular structure and nearly zero endogenous tissue autofluorescence. Furthermore, as shown in Fig. 3D and F, the SBR of the major abdominal vessel and femoral vessel in NIR-IIa imaging reached a value of 2.2 and 5.5, respectively. But the SBR of NIR-IIb imaging in the abdominal region (6.1) and femoral region (10.2) was 2.8-fold (P < 0.05) and 1.9-fold (P < 0.05) higher than that of NIR-IIa imaging, respectively. In particular, the direct measurement (DM) of the epigastric artery and femoral artery (Additional file 1: Fig. S2) was performed to determine the proportion of full width at half-maximum (FWHM) to DM (185 μm-epigastric artery, 618 μm-femoral artery). As shown in Fig. 3I and K, the epigastric artery and femoral artery diameter proportion of FWHM to DM in NIR-IIb imaging was closer to 1 than that in NIR-IIa imaging (P < 0.05). These results demonstrated that the NIR-IIb imaging could offer higher resolution imaging performance than NIR-IIa imaging, which suggested the potential of RNase A@PbS/ZnS QDs serving as an optimal nanoprobe to provide more accurate theranostic information in precision medicine.

Nowadays, it is well known that the flap perfusion directly correlates with the chances of survival of translated flap and surgical prognosis. The superior thermal stability and vascular imaging performance encouraged us to further explore the potential of RNase A@PbS/ZnS QDs for long time visualization of the microvasculature of the flap perfusion, which could assist the precise assessment of necrosis and survival areas based on our previous works on flap transplantation [28]. To build flap transplantation animal model, a 4.5 cm × 2.5 cm dorsal island perforator skin flap was measured and elevated with the left deep circumflex iliac artery perforator as a pedicle according to our previous stabled method [28]. The mice were intravenously injected with 200 μL of RNase A@PbS/ZnS QDs at a concentration of 1.5 mg/mL (corresponding to a dose of 15 mg/kg). The present dose was slightly lower than what was previously reported for (PbS)/CdS QDs (2 mg/mL) and was consistent with the dose of Ag_2_S QDs (2 mg/mL) [12, 29]. The total volume remained the same.

**Fig. 4:**
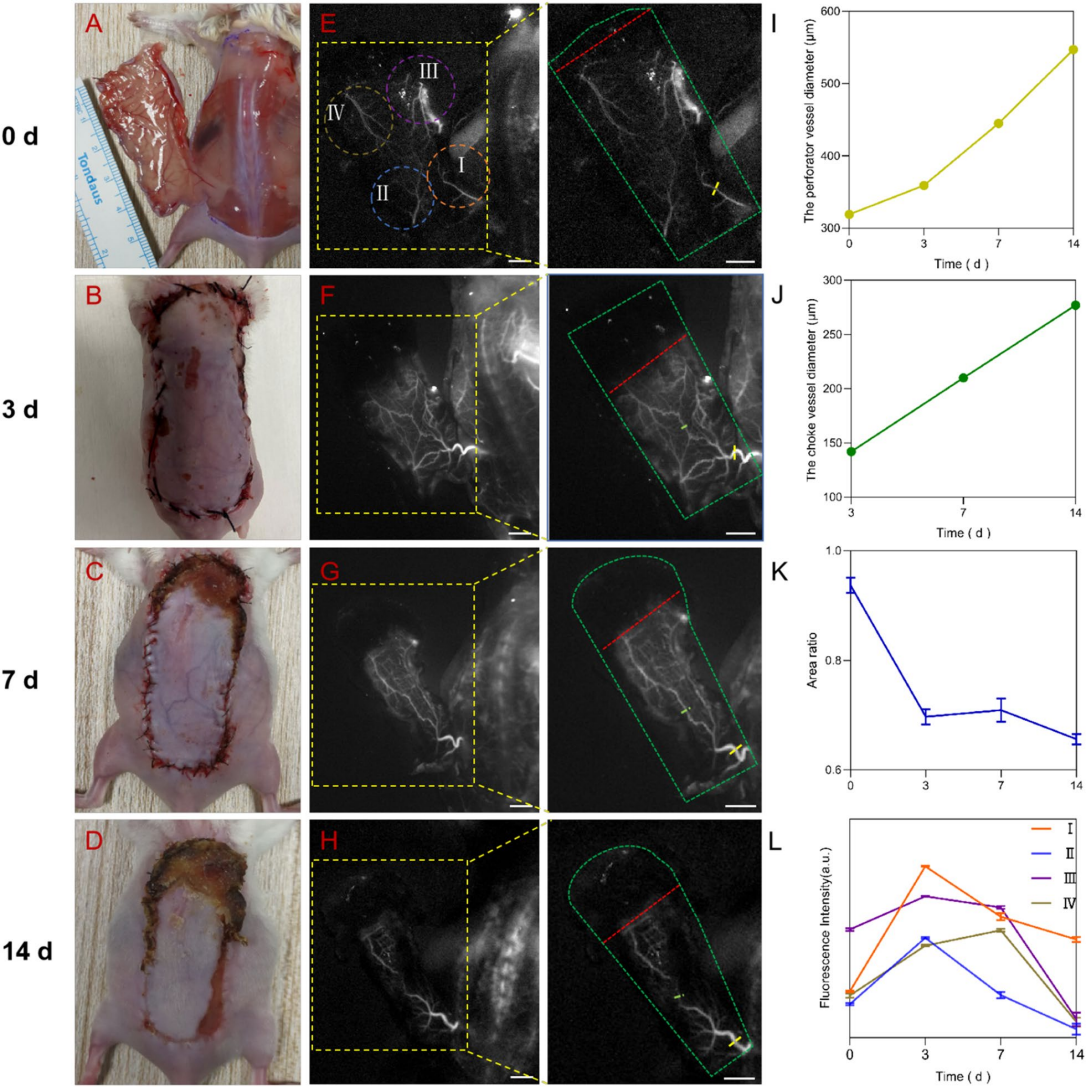
In vivo visualizing the microvasculature map of the flap. **A**–**D** Time course of bright field images in a flap perfusion defect animal model at different time points after injection of RNase A@PbS/ZnS QDs. **E**–**H** Time course of corresponding NIR-II fluorescence images. The area of the perforator flap was marked by the green dotted line, and the red dotted line marked the boundary between the bright zone and the dark zone; the yellow dashed line indicated the perforator vessel and the green dashed line indicated the choke vessel. Quantitative measurement of the diameter of the perforator vessel (**I**) and the choke vessel (**J**). **K** The area ratio between bright zone and total zone. **L** The fluorescence intensity analysis of the four perforasome

The appearance of perforator flap at 0, 3, 7, 14 days post-operation is showed in Fig. 4A–D, and the dark zone rose with time, which would be the necrosis areas based on our previous study. As shown in Fig. 4E–H, the blood vessels of the flap were clearly visible. Then Gaussian fit was employed to measure the diameter of perforator vessel and choke vessel at different time. As shown in Fig. 4I, the Gaussian­fit diameter of the left deep circumflex iliac artery perforator increased over time. Meanwhile, the Gaussian­fit diameter of the choke vessel in the center of the flap also increased from 3 to 14 days post-operation (Fig. 4J). In addition to blood vessels, the bright zone is also an important index to evaluate the perforator flaps. The area ratio of the bright zone to the total zone decreased from 0 to 3 days and 7–14 days post-operation (Fig. 4K). The fluorescence intensity of the perforasome I, II and III increased at the first 3 days and decreased from 3 to 14 days post-operation (Fig. 4L). The fluorescence intensity of the perforasome IV increased at the first 7 days and decreased from 7 to 14 days post-operation. In conclusion, NIR-IIb fluorescence imaging validated the capacity of the as-prepared RNase A@PbS/ZnS QDs for long time visualization of the flap perfusion in a flap transplantation animal model with high-resolution. The high-resolution of NIR-IIb vascular imaging contributes to enhance intraoperative blood vessel visualization, and paves the way for improving the survival rate of flap transplantation after surgery.

Although, the biocompactility of RNase A based QDs have been widely studied in our previous works [27, 30, 31], further systemic investigations were required to understand the pharmacokinetics and potential toxicity of RNase A@PbS/ZnS QDs for potential clinical application. As shown in Additional file 1: Fig. S4A, all the mice were alive and there was no obvious difference in body weight between the RNase A@PbS/ZnS QDs–treated mice and the healthy mice. Furthermore, the blood circulation half-life of RNase A@PbS/ZnS QDs was more than four hours, suggesting its convenience and appeal in a continuous imaging-guided diagnosis and treatment (Additional file 1: Fig. S4B). In addition, the blood, feces and major organs were collected at predetermined time points to measure the Pb^2+^ concentration using inductively coupled plasma atomic emission spectroscopy (ICP-AES). The clearance of RNase A@PbS/ZnS QDs was further examined and it was found that the amount of Pb^2+^ cleared through feces increased quickly during 24 h post-injection and followed by a gradual decrease (Additional file 1: Fig. S4C). About 69% of injected RNase A@PbS/ZnS QDs were excreted within 21 days after injection. Furthermore, the main organs of mice were collected at 21 days after injection to quantify the in vivo biodistribution of RNase A@PbS/ZnS QDs after leaving the bloodstream. Little to no retention of Pb^2+^ was observed in major organs while liver and spleen were the dominant organs for accumulating RNase A@PbS/ZnS QDs at 1.05 and 0.9 percentage of injection dosage per gram of tissue, respectively (Additional file 1: Fig. S4D). As shown in Additional file 1: Fig. S5A, B, most of the organs, except for the liver and spleen, showed subtle NIR-IIb signal at 21 days post-injection. The higher concentration of the QDs accumulated in liver and spleen could be attributed to the higher uptake of RNase A@PbS/ZnS QDs owing to the reticuloendothelial system after intravenous injection. Meanwhile, the accumulation in kidney displayed a low level possibly owing to a bit large diameter of RNase A@PbS/ZnS QDs, which was bigger than the renal filtration cutoff (5.5-nm) and prevented renal excretion from the body. Besides, no obvious injury or inflammation was observed in major organs (Additional file 1: Fig. S6). These results showed that RNase A@PbS/ZnS QDs exhibited excellent biocompatibility and low cyctotoxicity at the imaging dosage. It was also encouraging that the injected RNase A@PbS/ZnS QDs were cleared from the main organs and excreted from the body through the biliary pathway.

**Fig. 5:**
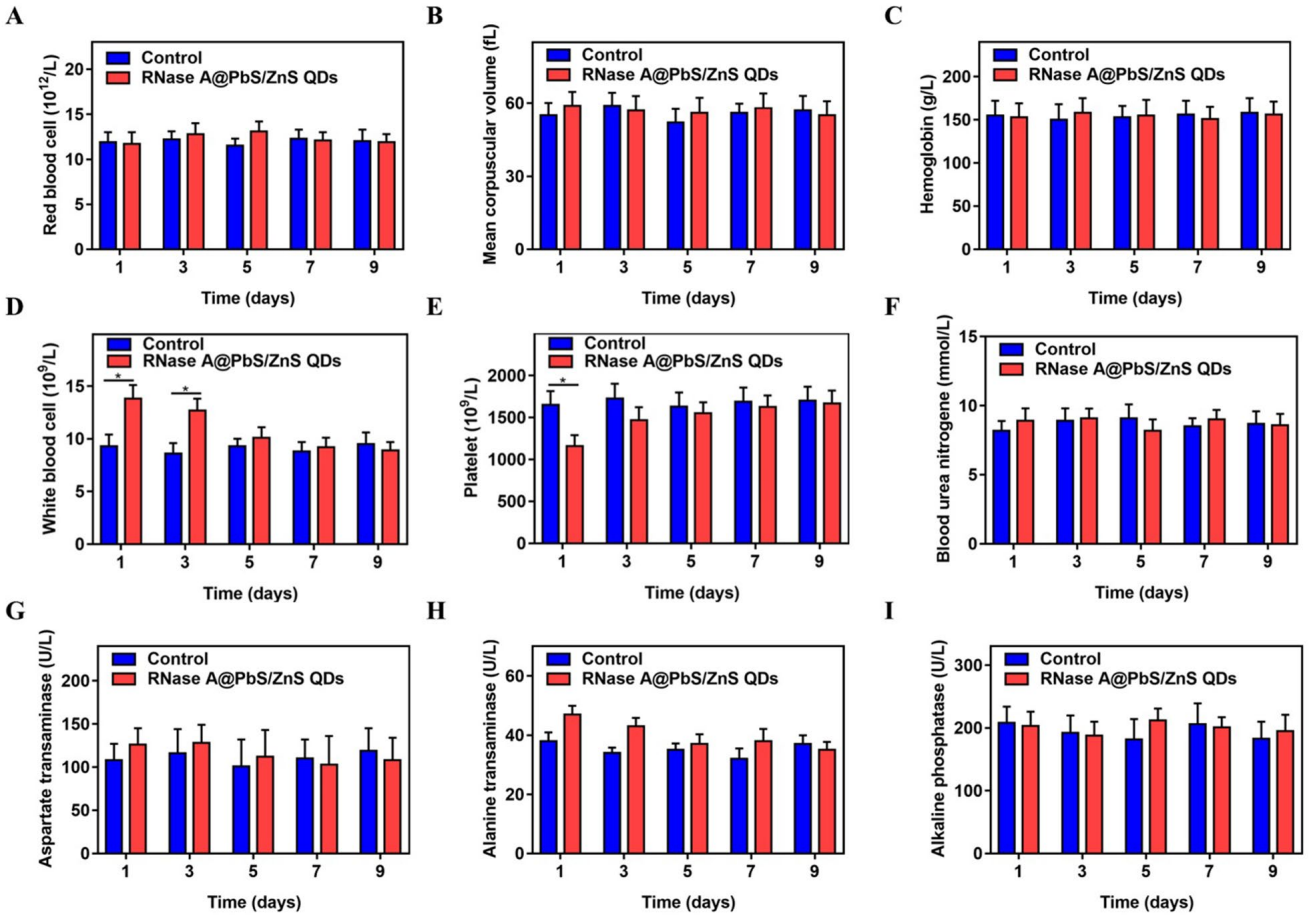
Blood biochemistry and hematology analysis of mice injected with RNase A@PbS/ZnS QDs at different time points. Mice treated with PBS solution served as control. **A** Red blood cell count analysis. **B** Mean corpuscular volume analysis. **C** Hemoglobin analysis. **D** White blood cell count analysis. **E** Platelet count analysis. **F** Blood urea nitrogen analysis. **G** Aspartate aminotransferase analysis. **H** Alanine aminotransferase analysis. **I** alkaline phosphatase analysis. Statistical analysis was based on three mice per data point. *P < 0.05

Although most of RNase A@PbS/ZnS QDs were excreted from the body with feces, further investigation of the in vivo toxicity will undoubtedly assist in improving their biological application. Therefore, blood biochemistry analysis and hematology analysis were performed to reveal potential toxicity. The results demonstrated that the hematological markers, except for the white blood cell counts and platelets, presented no statistical difference between RNase A@PbS/ZnS QDs treated mice and control mice. The white blood cell level in the RNase A@PbS/ZnS QDs treated mice showed a slight rise while the platelet level demonstrated a slight decline during the first few days after the QD injection (Fig. 5). These early reactions are regarded as the result of macrophage inhibition and acute stress reactions, respectively [29]. Both levels gradually returned to normal in the following days. In addition, the hepatic and renal functions were further evaluated. As shown in Fig. 5, the blood urea nitrogen level in the RNase A@PbS/ZnS QDs treated mice kept stable within normal limits after the QD injection, suggesting the good working order of the kidney. In particular, the liver function markers demonstrated no statistical difference between RNase A@PbS/ZnS QDs treated mice and control ones. Although the level of aspartate aminotransferase and alanine aminotransferase rose slightly during the first few days after injection, they were sustained in a normal range (Fig. 5G, H). This fluctuation might be attributed to the accumulation of RNase A@PbS/ZnS QDs in the liver. Therefore, we concluded that no appreciable toxicity of RNase A@PbS/ZnS QDs was detected in the mice at the present dosages.

## Conclusions

In conclusion, we successfully developed a superior thermally stable NIR-IIb fluorescence nanoprobe for precise detection of microvasculature network of the flap with high spatial resolution. The as-prepared RNase A@PbS/ZnS QDs offered several salient features: (1) due to the encapsulation of RNase A, the water-soluble RNase A@PbS/ZnS QDs displayed their impressive photostability and biocompatibility; (2) the RNase A@PbS/ZnS QDs exhibited superior thermal stability and reduced the concern of the signal attenuation caused by thermal accumulation, rendering the long time use of lasers less of a concern; and (3) compared with NIR-IIa fluorescence imaging, NIR-IIb fluorescence imaging based on RNase A@PbS/ZnS QDs greatly improved imaging penetration depth and SBR, and provided precise flap necrosis regions detection in long-time imaging. Therefore, RNase A@PbS/ZnS QDs is a promising NIR-IIb fluorescence agent candidate for long-time intraoperative fluorescence image-guided surgery with superior spatial resolution, deep penetration and high imaging SBR, especially for flap transplantation surgery.

## Materials and methods

### Preparation of hydrophilic of RNase A@PbS/ZnS QDs

A 10 mM Pb(OAc)_2_ solution was used to prepare PbS QDs as described in the previous study [27]. A 500 μL portion of PbS QDs solution was added drop by drop into the mixture of 500 μL RNase-A (mg/mL) and 500 μL Zn(CH3COO)2 (5 mM) precursor solution. And NaOH solution (0.17 M) was used to basify the system pH to ~ 11. After stirring, 60 μL of 10 mM Na_2_S solution was added, then the mixed solution was heated at 70 °C for 60 s in a microwave reactor with an input power of 30 W.

### Characterizations

The NIR-II fluorescence spectra of RNase A@PbS/ZnS QDs were collected on an Applied NanoFluorescence spectrometer (USA) at room temperature with an excitation laser source of 658 nm. The quantum yield of RNase A@PbS/ZnS QDs were determined using the IR-26 dye dissolved in 1,2-dichloroethane as described in the previous study. The photostability of RNase A@PbS/ZnS QDs was detected after continuous irradiation with an 808 nm laser (Starway Laser Inc., China, 0.2 W/cm^2^). The fluorescence intensity change of RNase A@PbS/ZnS QDs was determined using the same fluorescence spectrometer during 4 weeks storage under room temperature. The TEM, HR-TEM and HAADF-STEM images of as-prepared RNase A@PbS/ZnS QDs were examined by a Tecnai F20 TEM (FEI, USA) operated at 200 kV. 1% Intralipid has a scattering effect similar to that of biological tissues, and is a common tool for simulating and evaluating the penetration depth and scattering effect of imaging probes in biological tissues [6, 7, 32, 33]. Capillary glass tubes filled with PbS QDs or RNase A@PbS/ZnS QDs were then placed under a cylindrical culture dish and covered with different volumes of 1% Intralipid solution. The depth of the capillary was calculated from the known area of the dish [6]. The entire device is excited by a 808 nm diode laser and emitted light was filtered through an 1000 nm long-pass filter. The fluorescence was captured by a 2D InGaAs detector and images were collected.

### Evaluation the cytotoxicity of RNase A@PbS/ZnS QDs

RNase A@PbS/ZnS QDs were conjugated to Tat peptide through covalent bonding by using the cross linking reagent Sulfo-SMCC (Sigma) as described in the previous study [30, 34]. MSCs were incubated with α-MEM medium containing Tat-RNase A@PbS/ZnS QDs (0, 10 µg/mL, 20 µg/mL, 30 µg/mL) for 12 h. The live/dead staining assay was performed at 7 days after incubation. Briefly speaking, the MSCs were incubated in calcein AM (2 × 10^−6^ M, staining live cells) and PI (8 × 10^−6^ M, staining dead cells) for 30 min at 37 °C and washed again with PBS. A fluorescence microscope (Olympus, Japan) was used to image and the images were further processed by Image J software to count viable cells.

### Detection of the thermal stability of RNase A@PbS/ZnS QDs

A fluorescence spectrometer was used to measure the fluorescence intensity changes of RNase A@PbS/ZnS QDs with temperature tuned from 20 to 60 °C. Six-week-old female Kunming mice were purchased from Shanghai Jiesijie Laboratory Animal Corporation. Animal studies were performed under the guidelines approved by Fudan University. Mice were anesthetized after intraperitoneal injection of 5% chloral hydrate (0.08 mL/10 g), and then the RNase A@PbS/ZnS QDs (5 mg/mL, 200 µL) were injected into mice through the tail vein, respectively. The mice were thermally imaged by an infrared thermal imager at excitation wavelengths of 808 nm (0.2 W/cm^2^) or 1064 nm (0.25 W/cm^2^). Images were collected and imported into IRBIS 3 software to analyze the mice body temperature change and record the highest temperature before injection and at 1, 2, 3, 4, 5, 10, 20, 30, 60 and 120 min after injection of RNase A@PbS/ZnS QDs. We studied the photothermal effects by recording maximum and minimum temperature changes using thermal imaging camera.

### In vivo fluorescence imaging in the NIR-II window

Mice were anesthetized after intraperitoneal injection of 5% chloral hydrate (0.08 mL/10 g), and then PbS QDs (1 mg/mL, 200 µL) or RNase A@PbS/ZnS QDs (5 mg/mL, 200 µL) were injected into mice through the tail vein, respectively. All NIR-II images were collected on a home-built NIR in vivo imaging system equipped with an InGaAs/shortwave infrared (SWIR) CCD camera (Photonic Science, UK) under an 808 nm diode laser (0.2 W/cm^2^). The emitted fluorescence from QDs was directed onto the InGaAs camera through an emission filter set (1000 nm long-pass filters, 1400 nm short-pass and 1500 nm long-pass filters) to select the NIR-IIa and NIR-IIb windows respectively. The exposure time was 100 ms. Finally, we compared and analyzed the SBR and epigastric artery and femoral artery FWHM to DM in the abdominal region and femoral region of mice.

### Flap transplantation animal model

The establishment of the animal model was based on our previously reported method with some modifications [28]. An 8-week-old ICR female mouse was anesthetized and placed in the prone position. Dorsal hair was removed with depilatory cream, and the surgical area was disinfected with 75% alcohol. A 4.5 cm × 2.5 cm dorsal island perforator skin flap was measured and elevated with the left deep circumflex iliac artery perforator as a pedicle. The flap contained skin, subcutaneous tissue, and panniculus carnosus. The flap was turned over from the left and attention was paid to the preservation of the left deep circumflex iliac artery perforator. The mouse was intravenously injected with 0.4 mL QDs. After NIR-II imaging, the flap was sutured using a 6/0 or 3/0 nylon. The fluorescence of a perforasome [35], the vascular territory of a single perforator, was measured and analyzed.

### In vivo pharmacokinetics and toxicity analysis of RNase A@PbS/ZnS QDs

The weight of the mice was measured at 1, 3, 5, 7, 9, 11, 13, 15, 17, 19, and 21 days after injection of RNase A@PbS/ZnS QDs. Blood was collected from the postorbital venous plexus of each mouse at predetermined times (1, 3, 5, 11, 17 and 21 h after injection). The blood samples were centrifuged and supernatant (plasma) was subsequently analyzed for Pb^2+^ concentration by inductively coupled plasma atomic emission spectroscopy (ICP-AES) as previous report [30]. In addition, metabolism cages were used to collect feces at 0.5, 1, 2, 3, 4 and 5 day after injection. The major organs of mice were collected and embedded in paraffin and cut into 5 µm sections and then stained with hematoxylin and eosin staining. Images were obtained by using an Olympus microscope. Blood biochemistry and hematology analysis were performed at 1, 3, 5, 7 and 9 days after intravenous injection of RNase A@PbS/ZnS QDs solutions. Mice treated with 200 μL of PBS solution served as control. The blood samples were separated into blood cells and blood plasma after a centrifugation of 3000 rpm for 20 min. The blood biochemistry and hematology analysis were performed at Shanghai Lab Animal Research Center.

### Statistical analysis

Quantitative data are expressed as the mean ± standard error of at least three determinations. Nonparametric data were analyzed with the Kruskal–Wallis test. Parametric data were compared by analysis of variance and the Tukey post hoc test. All statistical analyses were performed with SPSS 23.0 software. A P value < 0.05 was considered to be statistically significant.

## Supplementary Information

**Additional file 1:** **Fig. S1.** Characterization of NIR-IIb–emitting RNase A@PbS/ZnS QDs. (A) Absorption spectrum of RNase A@PbS/ZnS QDs. (B) Energy-dispersive X-ray of RNase A@PbS/ZnS QDs. (C) Selected area electron diffraction pattern of RNase A@PbS/ZnS QDs. (D) TEM images of RNase A@PbS/ZnS QDs. (E) Size-distribution of RNase A@PbS/ZnS QDs. **Fig. S2.** The effects of different concentrations of RNase A@PbS/ZnS QDs on MSCs viability. (a). Live/dead cell staining for MSCs (green fluorescence-live cells, red fluorescence-dead cells). Scale bars represent 50 μm. (b). Quantification analysis of MSCs viability. **Fig. S3.** DM the diameter of epigastric artery (A) and femoral artery (B); the blue arrows indicate the femoral artery, and the yellow arrows indicate the epigastric artery. **Fig. S4.** In vivo pharmacokinetics and biodistribution of RNase A@PbS/ZnS QDs in normal mice. (A) Body weight of RNase A@PbS/ZnS QDs treated mice over a period time of 21 d. (B) Time course of Pb^2+^ concentration in the blood of RNase A@PbS/ZnS QDs treated mice over 21 h. (C) Time course of Pb^2+^ concentration in the feces of RNase A@PbS/ZnS QDs treated mice. (D) Biodistribution of Pb^2+^ in organs. **Fig. S5.** In vivo biodistribution of RNase A@PbS/ZnS QDs in flap perfusion animal model mice (A) Bright field and NIR-IIb fluorescence images of various organs collected from the mice at 21 days after postinjection. (B) Quantitative analysis NIR-IIb signal intensity of various organs. **Fig. S6.** Representative photomicrographs of hematoxylin and eosin staining on the major organs of the mice after injection of RNase A@PbS/ZnS QDs.

## Data Availability

All data generated or analysed during this study are included in this published article and its additional files.
