# Supplementary material for: Highly thermal stable RNase A@PbS/ZnS quantum dots as NIR-IIb image contrast for visualizing temporal changes of microvasculature remodeling in flap
Source: J Nanobiotechnology. 2022 Mar 12;20:128. doi: 10.1186/s12951-022-01312-0 (PMC8917748; doi:10.1186/s12951-022-01312-0)
Supplement: Supplementary file 1 — Additional file 1: Fig. S1. Characterization of NIR-IIb–emitting RNase A@PbS/ZnS QDs. (A) Absorption spectrum of RNase A@PbS/ZnS QDs. (B) Energy-dispersive X-ray of RNase A@PbS/ZnS QDs. (C) Selected area electron diffraction pattern of RNase A@PbS/ZnS QDs. (D) TEM images of RNase A@PbS/ZnS QDs. (E) Size-distribution of RNase A@PbS/ZnS QDs. Fig. S2. The effects of different concentrations of RNase A@PbS/ZnS QDs on MSCs viability. (a). Live/dead cell staining for MSCs (green fluorescence-live cells, red fluorescence-dead cells). Scale bars represent 50 μm. (b). Quantification analysis of MSCs viability. Fig. S3. DM the diameter of epigastric artery (A) and femoral artery (B); the blue arrows indicate the femoral artery, and the yellow arrows indicate the epigastric artery. Fig. S4. In vivo pharmacokinetics and biodistribution of RNase A@PbS/ZnS QDs in normal mice. (A) Body weight of RNase A@PbS/ZnS QDs treated mice over a period time of 21 d. (B) Time course of Pb2+ concentration in the blood of RNase A@PbS/ZnS QDs treated mice over 21 h. (C) Time course of Pb2+ concentration in the feces of RNase A@PbS/ZnS QDs treated mice. (D) Biodistribution of Pb2+ in organs. Fig. S5. In vivo biodistribution of RNase A@PbS/ZnS QDs in flap perfusion animal model mice (A) Bright field and NIR-IIb fluorescence images of various organs collected from the mice at 21 days after postinjection. (B) Quantitative analysis NIR-IIb signal intensity of various organs. Fig. S6. Representative photomicrographs of hematoxylin and eosin staining on the major organs of the mice after injection of RNase A@PbS/ZnS QDs. [file 12951_2022_1312_MOESM1_ESM.docx]

**Additional file 1**

**Highly Thermal Stable RNase A@PbS/ZnS Quantum Dots as NIR-IIb Image Contrast for Long-time Intraoperative Imaging Navigation for Flap Transplantation**

Yimeng Yang ^1^**^†^**, Mo Chen^1^**^†^**, Peng Wang^1^**^†^**, Liman Sai^2^, Chen Chen^3^, Pingkang Qian^4^, Shixian Dong^5^, Sijia Feng^1^, Xing Yang^6^, Hao Wang^7^, Amr M Abdou^8^, Yunxia Li^1^, Shiyi Chen^1^, Yuefeng Hao^6*^, Dongling Ma^9*^, Shaoqing Feng^10*^ and Jun Chen^1*^

*****Correspondence: biochenjun@fudan.edu.cn; fmmufsq@163.com; [ma@emt.inrs.ca](mailto:ma@emt.inrs.ca); [13913109339@163.com](mailto:13913109339@163.com).

^†^Yimeng Yang, Mo Chen and Peng Wang contributed equally to this work.

^1^Sports Medicine Institute of Fudan Univerisity, Department of Sports Medicine and Arthroscopy, Huashan Hospital, Fudan University, Shanghai, 200040, China. ^2^Department of Physics, Shanghai Normal University, Guilin Road 100, Shanghai 200234, China. ^3^Department of Sports Medicine, Shanghai Sixth People’s Hospital Affiliated to Shanghai Jiao Tong University, Shanghai, 200233, China. ^4^Department of orthopedics, Kunshan Hospital of Traditional Chinese Medicine, West Chaoyang Road,Kunshan City, Jiangsu Province 215300, China. ^5^Department of Anatomy and Physiology, School of Medicine, Shanghai Jiao Tong University, Shanghai, 200025, China. ^6^Department of orthopedics, Affiliated Suzhou Hospital of Nanjing Medical University, Suzhou 215500, Jiangsu, China. ^7^Asia Cellular Therapeutics (Shanghai) Co., Ltd, Shanghai, 201499, China. ^8^Department of Microbiology and Immunology, National Research Center, Giza, Egypt. ^9^Institut National de la Recherche Scientifique Centre Énergie Matériaux et Télécommunications, Université du Québec, 1650 Boulevard Lionel-Boulet, Varennes, Quebec J3X 1S2, Canada. ^10^Department ofPlastic and Reconstructive Surgery, Shanghai Ninth People's Hospital, School ofMedicine, Shanghai JiaoTong University, Shanghai, 200011, China.

Additional Texts

Fig. S1. Characterization of NIR-IIb–emitting RNase A@PbS/ZnS QDs.

Fig. S2. DM the diameter of epigastric artery and femoral artery.

Fig. S3. In vivo pharmacokinetics and biodistribution of RNase A@PbS/ZnS QDs in normal mice.

Fig. S4. In vivo biodistribution of RNase A@PbS/ZnS QDs in flap perfusion animal model mice.

Fig. S5. Representative photomicrographs of hematoxylin and eosin staining on the major organs of the mice after injection of RNase A@PbS/ZnS QDs.


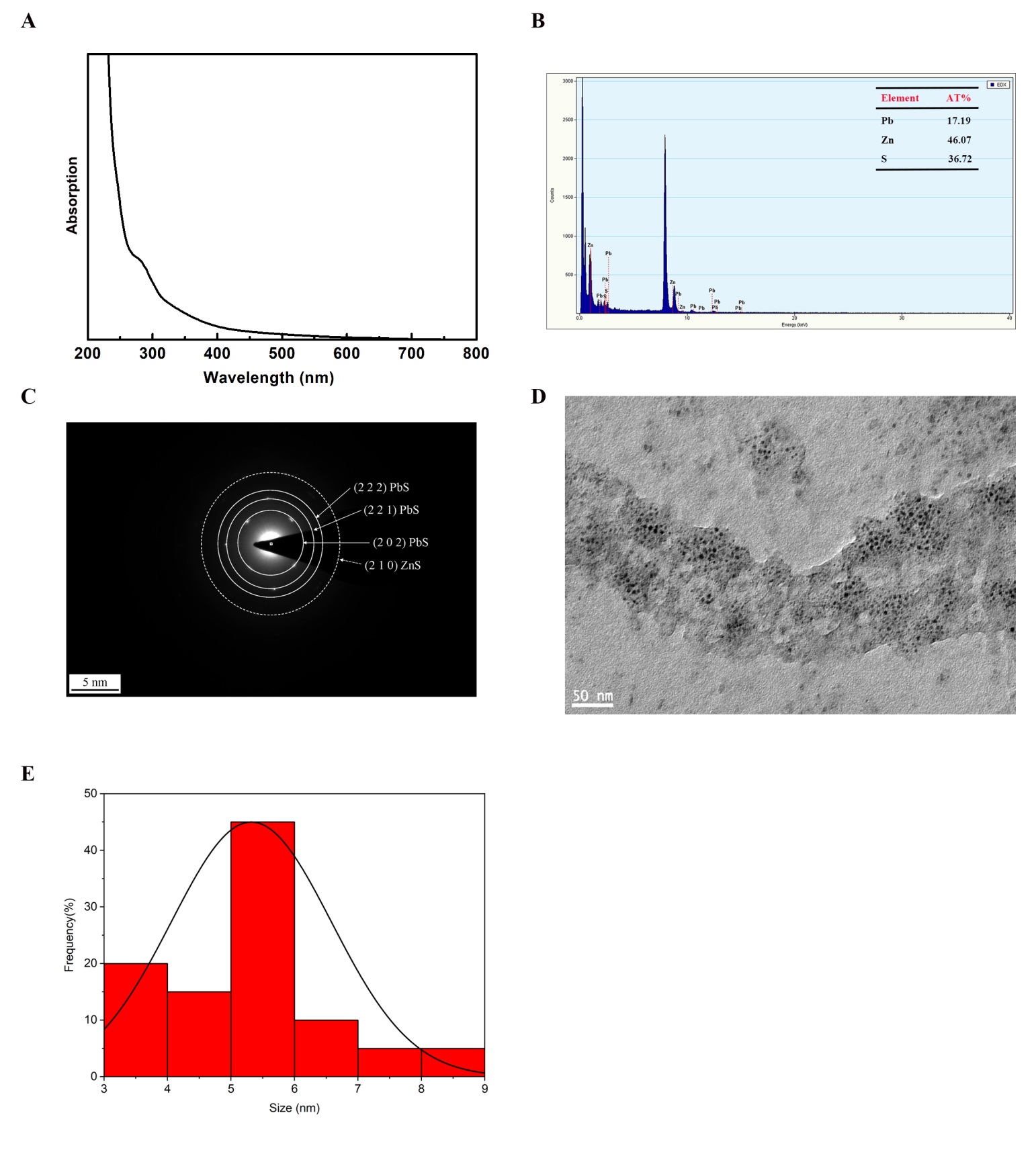


**Fig. S1.** Characterization of NIR-IIb–emitting RNase A@PbS/ZnS QDs. (A) Absorption spectrum of RNase A@PbS/ZnS QDs. (B) Energy-dispersive X-ray of RNase A@PbS/ZnS QDs. (C) Selected area electron diffraction pattern of RNase A@PbS/ZnS QDs. (D) TEM images of RNase A@PbS/ZnS QDs. (E) Size-distribution of RNase A@PbS/ZnS QDs.


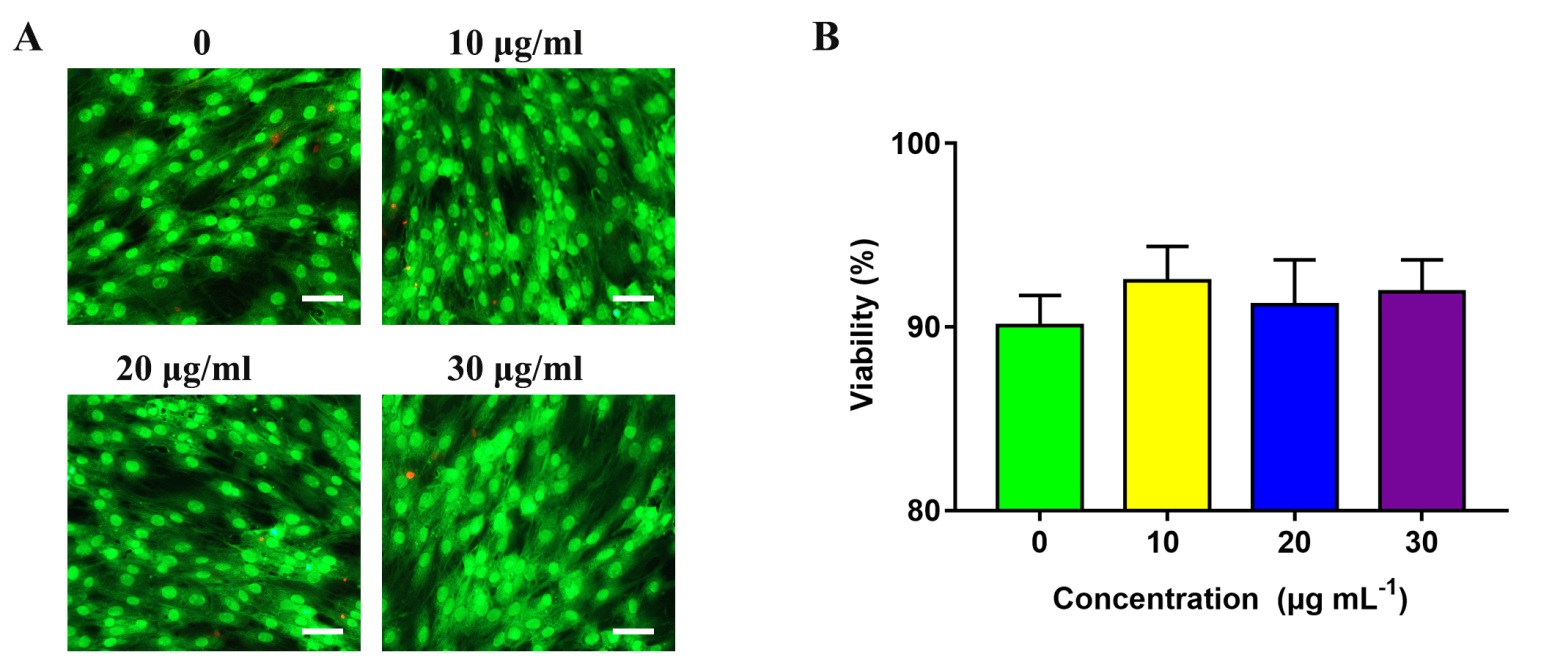


**Fig. S2.** The effects of different concentrations of RNase A@PbS/ZnS QDs on MSCs viability. (a). Live/dead cell staining for MSCs (green fluorescence-live cells, red fluorescence-dead cells). Scale bars represent 50 μm. (b). Quantification analysis of MSCs viability.


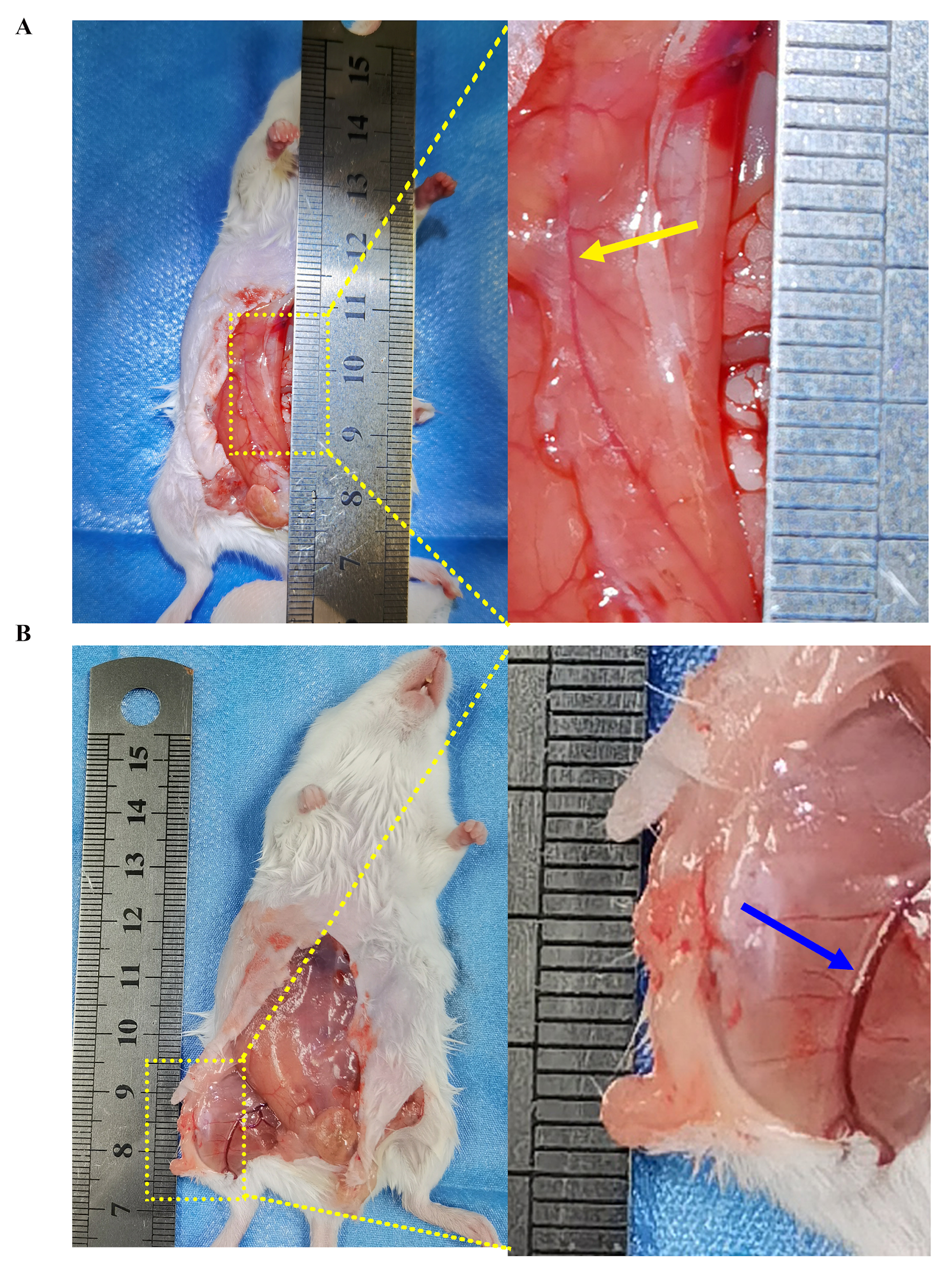


**Fig. S3.** DM the diameter of epigastric artery (A) and femoral artery (B); the blue arrows indicate the femoral artery, and the yellow arrows indicate the epigastric artery.


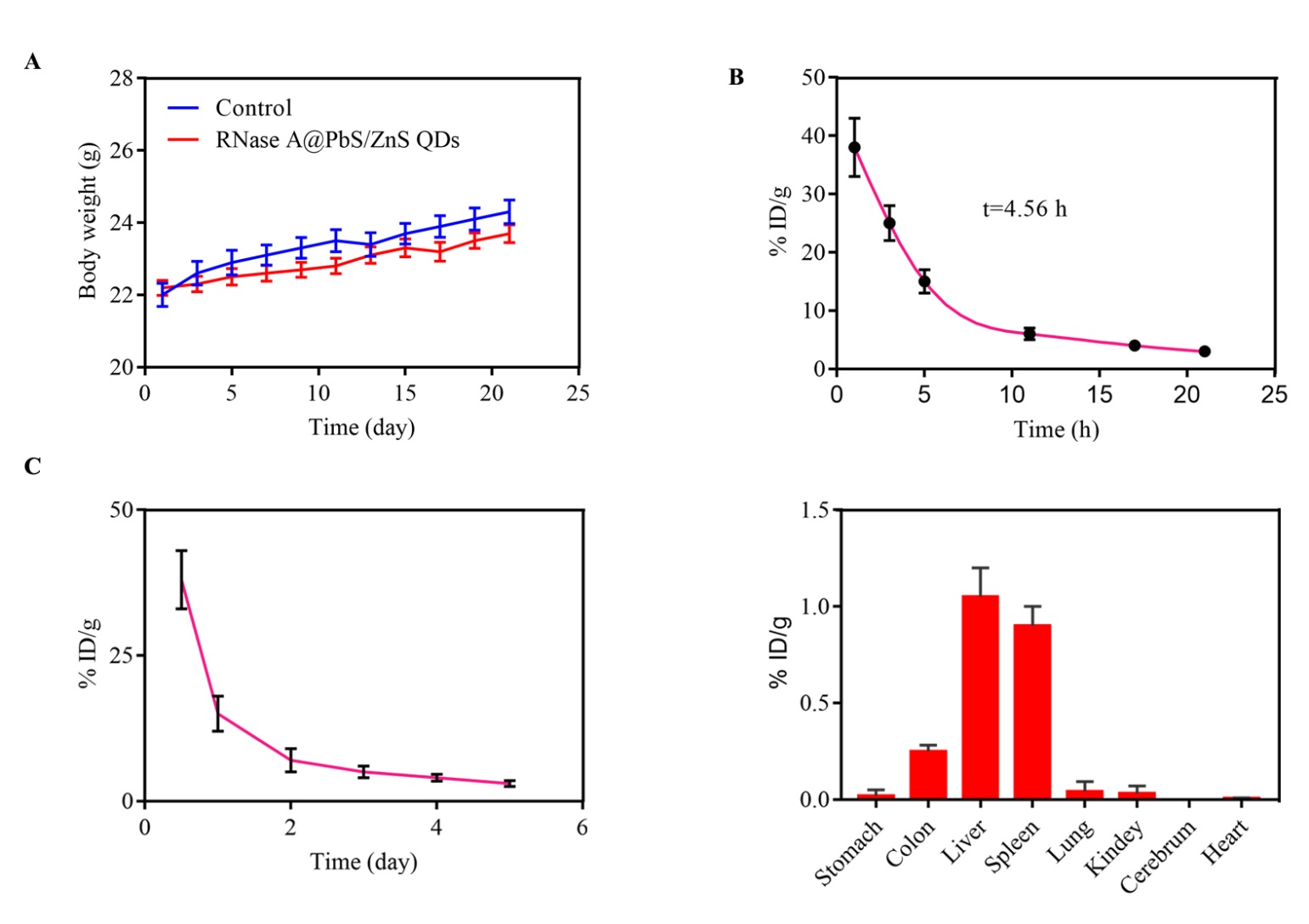


**Fig. S4.** In vivo pharmacokinetics and biodistribution of RNase A@PbS/ZnS QDs in normal mice. (A) Body weight of RNase A@PbS/ZnS QDs treated mice over a period time of 21 d. (B) Time course of Pb^2+^ concentration in the blood of RNase A@PbS/ZnS QDs treated mice over 21 h. (C) Time course of Pb^2+^ concentration in the feces of RNase A@PbS/ZnS QDs treated mice. (D) Biodistribution of Pb^2+^ in organs.


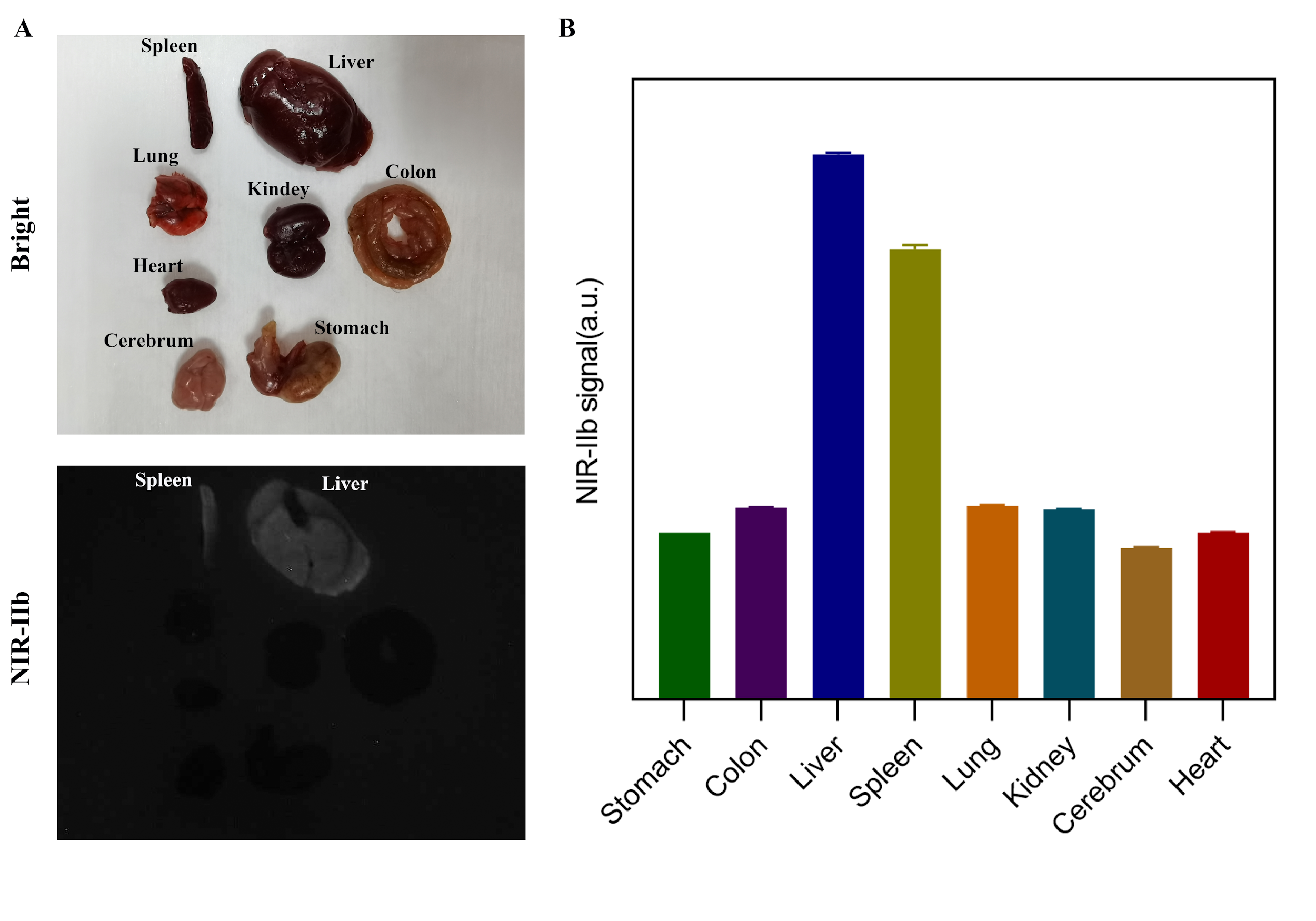


**Fig. S5.** In vivo biodistribution of RNase A@PbS/ZnS QDs in flap perfusion animal model mice (A) Bright field and NIR-IIb ﬂuorescence images of various organs collected from the mice at 21 days after postinjection. (B) Quantitative analysis NIR-IIb signal intensity of various organs.


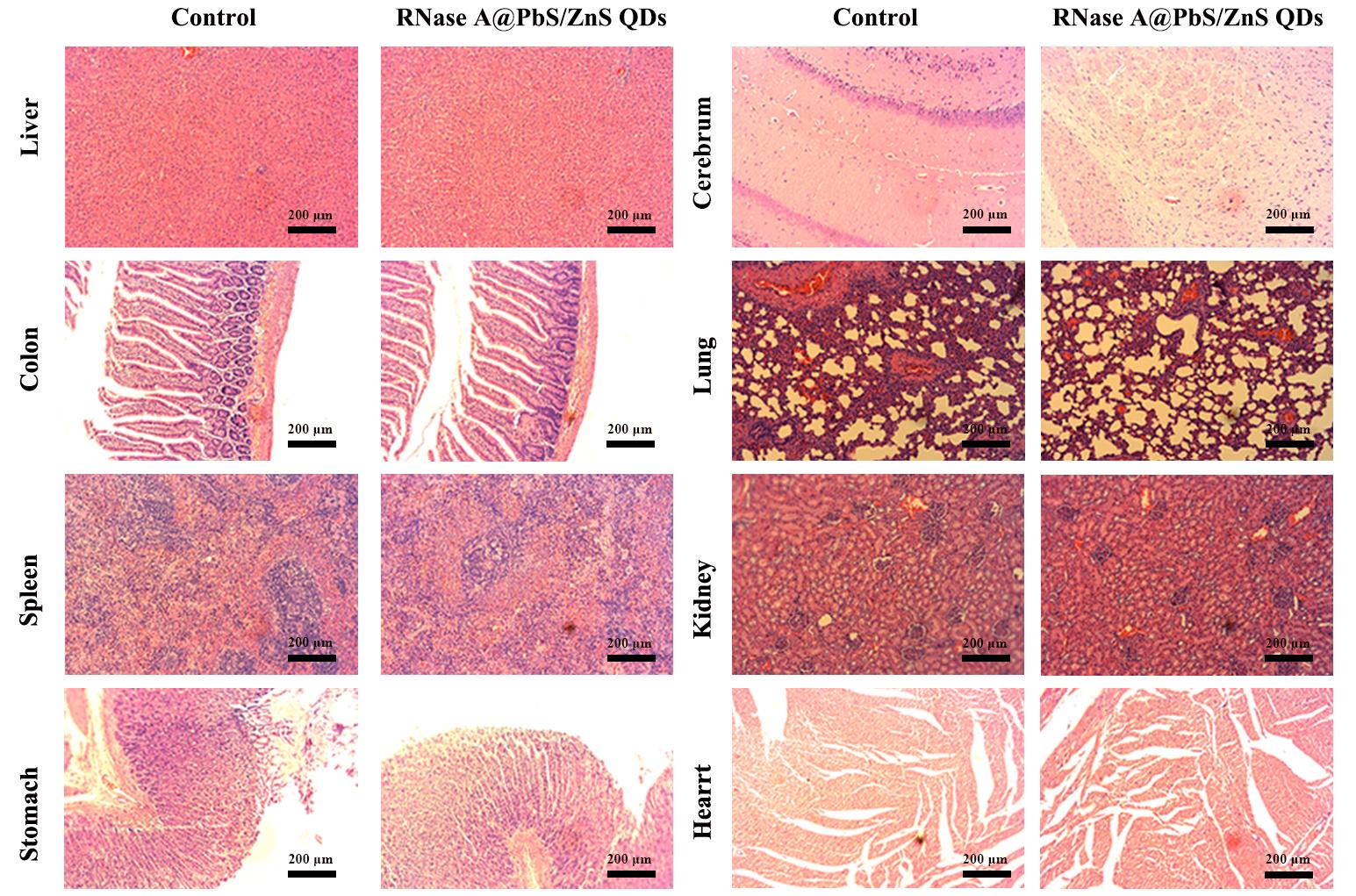


**Fig. S6.** Representative photomicrographs of hematoxylin and eosin staining on the major organs of the mice after injection of RNase A@PbS/ZnS QDs.
